# A comprehensive introductory course on systematic review and meta-analysis simultaneously delivered in-person and online in Cameroon

**DOI:** 10.1186/s13643-023-02212-6

**Published:** 2023-03-16

**Authors:** Jean Joel Bigna, Willy Steven Kamgang, Georges Nguefack-Tsague

**Affiliations:** 1 Department of Epidemiology and Public Health, Centre Pasteur of Cameroon, Yaounde, Cameroon; 2 Fishboard SARL, Yaounde, Cameroon; 3 Department of Public Health, Faculty of Medicine and Biomedical Sciences, University of Yaounde I, Yaounde, Cameroon

## Abstract

The global increased on research outputs around health research in general and systematic reviews has not left Sub-Saharan Africa indifferent, and Cameroon in particular. Research projects have not only increased in number but also in complexity with a record proportion of systematic review projects. This indicates that there is an unprecedented need to increase knowledge and expertise on systematic reviews and meta-analyses for high-quality evidence synthesis. We plan on associating local and international expertise to strengthen the planning, conduct and reporting of systematic reviews—in and out of academia—through an integrative learning program delivered both in English and French, the official languages in the country. The general of this introductory course was to assess the feasibility of our learning program as well as the extent to which the target audience would be willing to adhere to the program. Overall, there were 148 attendees, with 51 in-person and 97 online. Forty percent of in-person attendees were university lecturers at medical schools in Cameroon. Overall, we noted that the attendees were impressed with the quality of the presentation and the general organization of this pedagogical event, and are looking forward to participating in future courses.

## Introduction

In the last two decades, the number of systematic reviews and meta-analyses indexed in PubMed has increased from about 2157 in 2000 to about 28,785 in 2022 [1]. This global increased on research outputs around health research in general and systematic reviews in particular has not left Sub-Saharan Africa indifferent. In Cameroon, the increase in number of medical schools (from one in 2000 to seven in 2022) alongside the increase of the *numerus clausus* for physicians, pharmacists, and dentists (from 100 in 2000 to more than 800 in 2022) and the recent creation of doctoral schools within these medical schools has markedly intensified medical research activities in the country. Research projects have not only increased in number but also in complexity with a record proportion of systematic review projects. This indicates that there is an unprecedented need to increase knowledge and expertise on systematic reviews and meta-analyses for high-quality evidence synthesis. We plan on associating local and international expertise to strengthen the planning, conduct and reporting of systematic reviews—in and out of academia—through an integrative learning program delivered both in English and French, the official languages in Cameroon.

There are a number of factors that may favor the implementation of such a program in Cameroon and beyond. First, there is an ongoing political initiative to improve health governance through the promotion of training programs that aim at enhancing the data management and research skills of the healthcare personnel. Second, the Cameroonian diaspora is comprised of numerous world class health researchers that are willing to share their knowledge and experience. Third, there is good internet penetration in most cities in Cameroon which can allow us to scale the impact of this educative program in an efficient and effective manner. Fourth, we intend to exempt participants from any financial charges related to tuition so that enrolment on the program would essentially be need-based.

As a first step toward the final draft of our educational program to reinforce research ability in systematic reviews, we delivered a free comprehensive introductory course on systematic reviews and meta-analysis—online and in-person.

## Objective of the course

The general objective of this introductory course was to assess the feasibility of our learning program as well as the extent to which the target audience would be willing to adhere to the programme.

## Approach

It was a 3-h course which took place on Saturday 13 August 2022 at the Study Room of *Fishboard SARL* in Yaounde with participants having the possibility to attend in-person or online through the Zoom app. *Fishboard SARL* is a private company based in the Emana District of Yaounde which promotes education and learning through a wide range of services. Its Study Room is a mini-conference hall that has been specially designed to host pedagogical events with Wi-Fi, up-to-date video-audio systems, and a premium Zoom subscription to enable active distance participation.

The event was officially announced one week earlier using a flyer and a 30-s invitation video (https://www.linkedin.com/feed/update/urn:li:activity:6961540496259223552) both in the English and French languages. These were posted on social media and allowed to be shared without boosting. We restricted in-person invitation to potential participants with initial training in research method including but not limited to university lecturers, post-doctoral fellows, PhD holders, students at the doctoral schools, public health professionals, residents in medicine, and specialist medical doctors. We verified the identity of all those who manifested their interest in participating in the event and saved their credentials onto an excel sheet.

It was a comprehensive course which covered introductory materials including an overview of current guidelines to write systematic review protocols and final reports, as well as many key references for further reads. The central idea was to provide an engaging presentation that would allow attendees to ask questions as the presentation unfolds. The course was totally free for all participants and no incentives were given in any form. The event was entirely funded by the organizers.

The course was recorded with the Zoom app and the recording was later on made freely available on YouTube. The PowerPoint presentation that was used was also emailed to all the participants who requested it.

## Outcome

Overall, there were 148 attendees, with 51 in-person and 97 online. Forty percent of in-person attendees were university lecturers at medical schools in Cameroon. The course effectively lasted about 4 h with over 2 h of follow-up interactions. The main reason for the increase in duration of the course was the numerous questions that were asked all through highlighting the highest interest of participants to the training.

The technical set up for remote participation was effective and permitted the event to be followed online with high-quality audio and video (video recording available on YouTube: https://www.youtube.com/watch?v=XqbTMkhzOhs&t=114s). Online participation did not only allow those in remote villages of Cameroon to participate, but also allowed us to have participants from other Sub-Saharan African countries such as Mali, Niger, and Burkina Faso.

Some attendees had specific questions on protocol writing for systematic reviews, suggesting that they were currently working on systematic review projects and needed pragmatic solutions to some problems they were facing. Others asked questions that were mostly related to basic definitions, suggesting that some participants had a vague knowledge about systematic reviews and needed more theoretical notions.

At the end of the course, we exchanged with the participants and collected the following major feedback:

- Some participants said that the course was too short to fully understand the concepts that featured in it. They wished that the course could last at least one full day and said that they were willing to pay for future courses.
- Although some appreciated the highly interactive nature of the presentation, some participants said that they would have preferred that all questions were dealt with in a question-and-answer session at the end of the presentation.
- Overall, we noted that the attendees were impressed with the quality of the presentation and the general organization of this pedagogical event, and are looking forward to participating in future courses.

## Data Availability

Not applicable.
